# A nomogram predicts cardiovascular events in patients with peritoneal dialysis-associated peritonitis

**DOI:** 10.1080/0886022X.2022.2126785

**Published:** 2022-09-26

**Authors:** Dan-dan Huang, Yuan-yuan Li, Xiang-ming Qi, Yong-gui Wu

**Affiliations:** Department of Nephropathy, the First Affiliated Hospital, Anhui Medical University, Hefei, Anhui, PR China

**Keywords:** Peritoneal dialysis-related peritonitis, risk factors, cardiovascular events, nomogram

## Abstract

**Objective:**

To predict the risk factors for cardiovascular events within 5 years in patients with peritoneal dialysis-associated peritonitis and establish a nomogram for clinical prediction.

**Methods:**

A prediction model was established by conducting an observational study in 150 patients with peritoneal dialysis-associated peritonitis obtained from the Information Database of AnHui Medical University Affiliated Hospital. The nomogram was constructed using the multivariate COX regression model. The C-index and the calibration plot were used to assess the discrimination and calibration of the prediction model.

**Results:**

The elderly [HR = 2.453 (1.071–5.619)], history of cardiovascular events [HR = 2.296 (1.220–4.321)], alkaline phosphatase [HR = 1.004 (1.002–1.005)] and culture-positive [HR= 2.173 (1.009–4.682)] were identified as risk predictors of cardiovascular events, while serum albumin [HR = 0.396(0.170–0.924)] was identified as protective predictors of cardiovascular events. Combined with clinical studies, we constructed a nomogram based on the minimum value of the Akaike Information Criterion or Bayesian Information Criterion. The C index of the nomogram is 0.732, revealing great discrimination and appropriate calibration. Through the total score of the nomogram and the result of ROC, we classify patients into high-risk groups (cardiovascular events group) and low-risk groups (no cardiovascular events group). Cardiovascular events were significantly different for patients in the high-risk group compared to the low-risk group (HR = 3.862(2.202–6.772; *p* < 0.001).

**Conclusions:**

The current novel nomogram can accurately predict cardiovascular events in patients with peritonitis associated with peritoneal dialysis. However, external validation is required before the model can be used in clinic settings.

## 1. Introduction

Cardiovascular events are common complications of chronic kidney disease, especially in the stage of end-stage renal disease; the incidence of cardiovascular events is about 15–20 times higher than that of the general population. Peritoneal dialysis (PD) is a preferred modality of replacement therapy for end-stage renal disease, has little effect on hemodynamics, preserves residual renal function, and reduces cardiovascular risk. However, the primary complication of PD is peritonitis. Recent studies have found increased cardiovascular mortality in patients with PD-associated peritonitis [1,2]. And further studies have confirmed that peritonitis is a risk factor for cardiovascular events and cardiovascular mortality in Australia [3–5].

The risk factors for cardiovascular events are numerous. The Framingham risk score is one of the most widely used tools to estimate the individual risk of cardiovascular events and has been validated in racially diverse general populations [6]. However, it is not suitable for patients with chronic kidney disease, especially those who require dialysis. A study on 201 hemodialysis patients aged 20–80 years reported that high-risk (>20% 10-year risk) classified by Framingham risk score cannot predict cardiovascular mortality [7].

Only limited data on predictive instruments for the risk of cardiovascular events in dialysis patients are available. An observational study to predict cardiovascular mortality in patients with PD included aging, history of cardiovascular disease, low hemoglobin and serum albumin, high-sensitivity C-reactive protein (CRP), and low 24 h urine output [8]. However, it is not suitable for predicting cardiovascular events in PD-associated peritonitis. First, the predicted crowds are different. Second, the purpose of the prediction is different. Cardiovascular mortality is not equal to cardiovascular events. Third, high-sensitivity CRP is susceptible to inflammation and needs to be reassessed. In the current study, our aim is to develop an accurate but simple prediction tool to predict risk factors for cardiovascular events after the first occurrence of peritonitis in patients with PD.

## 2. Methods

### 2.1. Study population

The current study enrolled consecutive incidents of initial peritonitis in adult patients reported from June 1, 2015 to May 31, 2021, at the AnHui Medical University Affiliated Hospital in China. All PD-related peritonitis met the 2016 ISPD diagnostic criteria [9]. All patients with PD-related peritonitis were treated with 2 L of lactated peritoneal dialysis solution, containing 1.5% glucose and 2.5% glucose. The peritoneal dialysis mode is CAPD. Patients with PD for <3 months, renal transplant, hemodialysis in 3 months, recovered renal function, lost follow-up, or initiated PD in another health service were excluded. Furthermore, patients who refused to provide written consent were excluded. Eligible participants gave their informed consent. The study was in accordance with the provisions of the Declaration of Helsinki (as revised in 2013) [10].

### 2.2. Variable selection

Clinical and laboratory data were obtained using standardized forms. Following variables were considered: the demographic variables, including age, sex, and smoking status; physical examination variables, including systolic blood pressure (SBP) and diastolic blood pressure (DBP), which were measured twice with a mercury sphygmomanometer in quiet conditions after half an hour of rest after admission to the hospital during the first onset of peritonitis, and the average was taken, heart rate, body weight with dry abdomen and height, which were also collected at first admission during the first onset of peritonitis. The body mass index was calculated according to weight and height. A history of cardiovascular events (CVEs) [11,12] was recorded, which was defined as a history of coronary heart disease, heart failure, stroke, and peripheral arterial disease. The data of laboratory variables were obtained at the first onset of peritonitis. Laboratory data, including white blood cells, neutrophil/lymphocyte ratio [13], hemoglobin, CRP, albumin, creatinine, serum uric acid, calcium, phosphorus, total triglycerides, high-density lipoprotein cholesterol (HDL-C), low-density lipoprotein cholesterol (LDL-C), intact parathyroid hormone (iPTH), alkaline phosphatase, 24 h urine output and culture results. Medication information, including angiotensin-converting enzyme inhibitor(ACEI)/angiotensin receptor blocker (ARB) therapy. PD information includes the duration of PD and peritoneal function, the first modality for renal replacement therapy [RRT], the number of peritonitis episodes during follow-up were measured at AnHui Medical University Affiliated Hospital. Other variables included diabetes and follow-up time. According to the time of onset of the first peritonitis, the peritonitis was divided into the early-onset peritonitis group (≤3 months) and the late-onset peritonitis group (>3 months).

### 2.3. Outcome

The primary outcome was a cardiovascular event defined as a combination of hospitalizations for coronary heart disease, heart failure, stroke, peripheral arterial disease, and sudden death, death associated with a cardiovascular procedure, death due to aneurysm dissection or rupture, fatal pulmonary embolism [14]. Heart failure means patients with a reduced ejection fraction (LVEF < 40%), diagnosed by echocardiography. Data on cardiovascular events were obtained from the National Center for Quality Medical Control of Nephrology registry in China and the coding of the 10th revision of the International Statistical Classification of Diseases and Related Health Problems (ICD-10). All patients were followed up until a cardiovascular event, renal transplant, hemodialysis therapy or the end of the study on 31 May, 2021.

### 2.4. Statistical analysis

Continuous variables are expressed as mean ± standard deviation or median (interquartile range) and were compared using the T or Mann-Whitney *U* test. Additionally, categorical variables were compared using Pearson’s *χ*^2^ or Fisher’s exact probability method test. Age, 24-h urine output, body mass index, and serum albumin were converted to categorical variables based on a routine cutoff point in clinical practice due to their skewed distribution. The rest of the variables were evaluated as linear predictors. All variables had less than 10% missing values, and all lost data were imputed using the miss Forest method, which is a nonparametric method for handling variables of different types simultaneously [15]. We selected the candidate predictors based on univariate regression analysis with a *P*-value less than 0.05. The stepwise selection was used to screen variables for multivariable COX regression analysis, combined with clinical data, we chose the minimum value of the Akaike Information Criterion and Bayesian Information Criterion, build the final model and constructed a nomogram based on it. In a multivariate analysis, the models were adjusted for demographic variables (age and gender; model 1), Framingham cardiovascular risk factors (age, sex, smoking, diabetes status, SBP, and HDL-cholesterol; model 2), factors associated with cardiovascular risk factors in patients with chronic kidney disease (CKD) (age, sex, diabetes status, CRP, phosphate, BMI, and serum albumin; model 3), and confounding factors associated with cardiovascular risk factors (alkaline phosphatase together with age and sex; model 4). Calculate the variance inflation factor (vif) for each variable to evaluate the collinearity of the final multivariate model. If vif is more than 10, consider the existence of collinearity. The Somers’ D correlation was also calculated to assess the calibration of the model. We used time-dependent receiver operating characteristic (ROC) analysis and area under the curve (AUC) to predict nomograms’ accuracy measured at different cutoff times. The score for each predictor in the nomogram was also calculated. Segmentation was performed using the optimal cutoff value determined by nomogram total score and ROC curve outcome when the Youden index was maximum, and patients were classified as 'low’ or 'high’ risk group. Kaplan-Meier curves were plotted for the two risk groups and survival was compared using the log-rank test curve. The C index and the calibration curve were used to determine the accuracy of the prediction and the discriminative power of the nomogram. A sensitivity analysis and a bootstrap with 1000 resampling was also performed to further determine the robustness of the model. Statistical tests were performed using R software version 3.0.1. The statistical significance was set at 0.05.

## 3. Results

### 3.1. Characteristics of study participants

**Figure 1.**
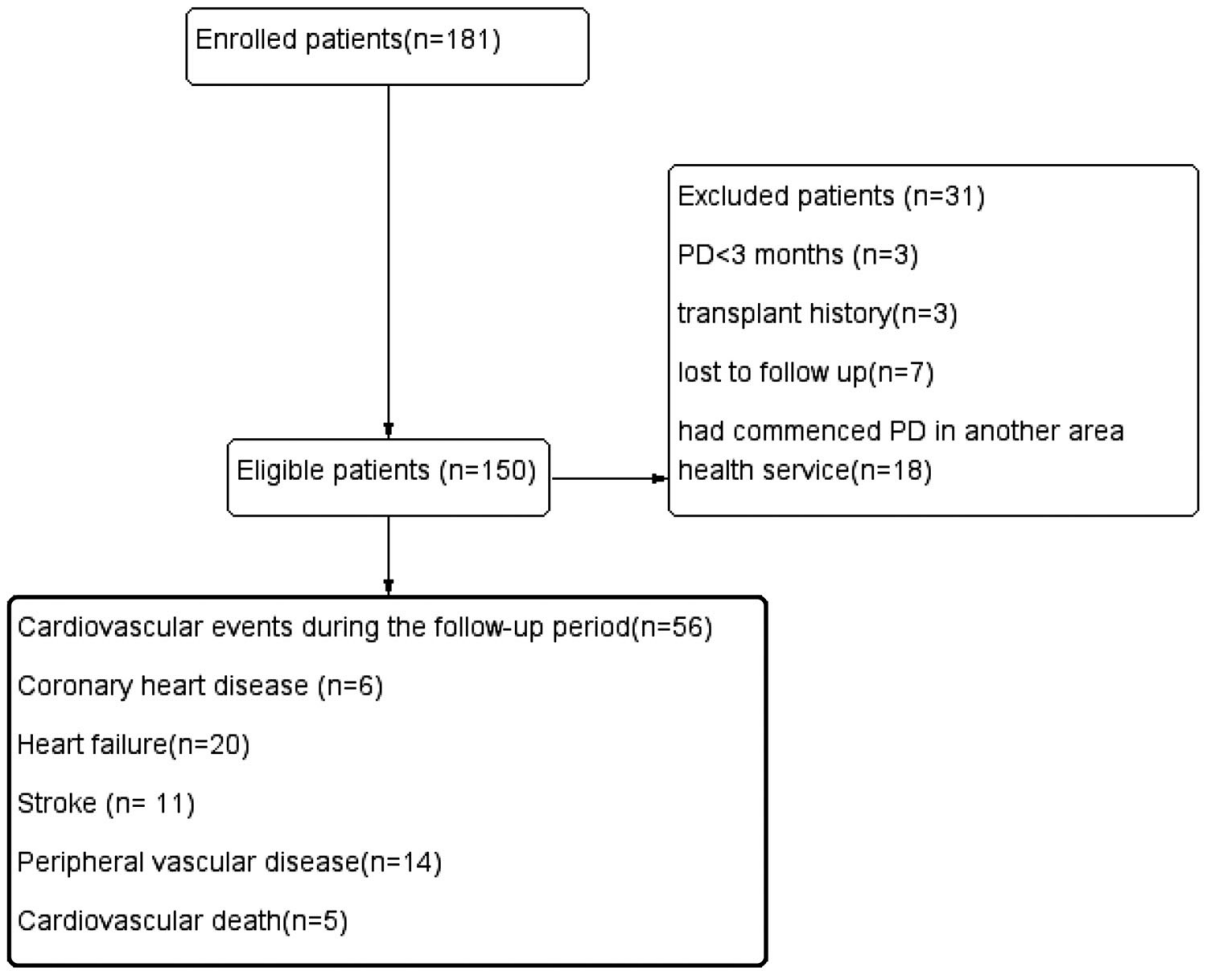
Enrollment and outcomes of the cohort.

In total, 150 eligible patients were included in the analysis and the mean follow-up time was 28 months. During follow-up, a total of 56 patients demonstrated cardiovascular events; 6 patients developed new coronary heart disease, 20 patients developed new heart failure, 11 patients developed new stroke, 14 patients developed the new peripheral vascular disease, and 5 patients developed cardiovascular death (Figure 1). The mean onset time of peritonitis was 38.49 months, more than the 60 months per patient anticipated by the International PD Association. The demographic characteristics, comorbidities, laboratory data, medication information, peritoneal dialysis information, and outcomes of the patients are listed in Table 1.

**Table 1.** Baseline characteristics of the study populations.

Variable	Total (*n* = 150)	0 (*n* = 94)	1 (*n* = 56)	*P*
Female	89 (59.33)	53 (56.38)	36 (64.29)	0.341
Age, year	51.02 ± 10.92	47.77 ± 9.40	56.48 ± 11.19	<0.001
Age.cut				<0.001
<65 years, *n*%	133 (88.67)	92 (97.87)	41 (73.21)	
≥65 years, *n*%	17 (11.33)	2 (2.13)	15 (26.79)	
BMI.cut(kg/m*m)				0.632
<18.5	10 (6.67)	6 (6.38)	4 (7.14)	
18.5–23.9	92 (61.33)	60 (63.83)	32 (57.14)	
24–27.9	37 (24.67)	23 (24.47)	14 (25.00)	
≥28	11 (7.33)	5 (5.32)	6 (10.71)	
Smoking, *n*%	17 (11.33)	11 (11.70)	6 (10.71)	0.854
Diabetes mellitus, *n*%	25 (16.67)	13 (13.83)	12 (21.43)	0.227
SBP, mmHg, ± sd	139.47 ± 24.09	136.13 ± 24.00	145.07 ± 23.39	0.027
DBP, mmHg, ± sd	90.13 ± 15.16	91.44 ± 15.10	87.95 ± 15.13	0.173
HR, times/min, ± sd	90.13 ± 15.25	90.72 ± 15.99	89.14 ± 14.01	0.541
History of CVEs	29 (19.33)	6 (6.38)	23 (41.07)	<0.001
White blood cells,10^9/L, ± sd	9.49 ± 3.96	9.71 ± 3.92	9.14 ± 4.02	0.769
Neutrophil/lymphocyte ratio,%, ± sd	11.56 ± 10.51	11.39 ± 11.28	11.83 ± 9.18	0.420
Hemoglobin, g/L, ± sd	95.75 ± 20.31	98.04 ± 19.47	91.91 ± 21.26	0.074
Creatinine, umol/L, ± sd	882.45 ± 253.54	913.69 ± 261.08	830.00 ± 233.28	0.050
Serum uric acid, umol/L, ± sd	374.80 ± 89.35	381.16 ± 87.85	364.12 ± 91.61	0.260
CRP, mg/L, ± sd	59.02 ± 54.69	56.77 ± 55.43	62.83 ± 53.70	0.517
Calcium, mmol/L, ± sd	2.26 ± 0.21	2.29 ± 0.21	2.20 ± 0.20	0.006
Phosphorus, mmol/L, ± sd	1.53 ± 0.57	1.55 ± 0.62	1.48 ± 0.48	0.484
Serum albumin, g/L				<0.001
<30, *n*%	46 (30.67)	21 (22.34)	25 (44.64)	
30 g–34.9, *n*%	47 (31.33)	25 (26.60)	22 (39.29)	
≥35, *n*%	57 (38.00)	48 (51.06)	8 (14.29)	
Alkaline phosphatase, u/L, ± sd	95.71 ± 100.50	82.33 ± 34.86	118.16 ± 156.49	0.034
Triglycerides, mmo/L, ± sd	1.57 ± 1.11	1.48 ± 0.93	1.71 ± 1.35	0.238
HDL-C, mmol/L, ± sd	1.15 ± 0.34	1.16 ± 0.32	1.15 ± 0.38	0.908
LDL-C, mmol/L, ± sd	2.74 ± 0.98	2.64 ± 0.94	2.91 ± 1.02	0.113
24hours urine output				0.913
<400, ml, *n*%	94 (62.67)	58 (61.70)	36 (64.29)	
400–1000, ml, *n*%	35 (23.33)	23 (24.47)	12 (21.43)	
>1000, ml, *n*%	21 (14.00)	13 (13.83)	8 (14.29)	
iPTH, pg/ml, ± sd	275.11 ± 285.31	265.18 ± 295.36	291.78 ± 269.38	0.582
ACEI/ARB, *n*%	46 (30.67)	29 (30.85)	17 (30.36)	0.949
Number of peritonitis episodes				0.482
≥3, *n*%	25 (16.67)	13 (13.83)	12 (21.43)	
1, *n*%	85 (56.67)	55 (58.51)	30 (53.57)	
2, *n*%	40 (26.66)	26 (27.66)	14 (25.00)	
Culture-positive, *n*%	108 (72.00)	60 (63.83)	48 (85.71)	0.004
The first modality for renal replacement therphy				0.478
PD, *n*%	143 (95.33)	91 (96.81)	52 (92.86)	
HD, *n*%	7 (4.67)	3 (3.19)	4 (7.14)	
Dialysis duration, month, ± sd	38.49 ± 32.33	38.29 ± 32.62	38.84 ± 32.11	0.920
Peritoneal function				0.097
Low transport, *n*%	14(9.59)	8(8.7)	6(11.11)	
Low average transport, *n*%	52(35.62)	39(42.39)	13(24.07)	
High average transport, *n*%	64(43.84)	38(41.3)	26(48.15)	
High transport, *n*%	16(10.96)	7(7.61)	9(16.67)	
Follow-up time, month, ± sd	28.60 ± 20.54	31.60 ± 21.06	23.57 ± 18.78	0.02

0: no cardiovascular events group; 1: cardiovascular events group.

BMI: body mass index; HR: heart rate; CVEs: cardiovascular events; SBP: systolic blood pressure; DBP: diastolic blood pressure; CRP: C-reactive protein; ACEI: angiotensin converting enzyme inhibitor; ARB: angiotensin receptor blockers; HDL-C, high density lipoprotein cholesterol; LDL-C, low-density lipoprotein cholesterol; iPTH, intact parathyroid hormone; PD: peritoneal dialysis; HD: hemodialysis; sd: standard deviation.

### 3.2. Univariate and multivariate COX regression analysis

Univariate COX analysis showed that age, age (≥65year), systolic blood pressure, history of CVEs, alkaline phosphatase, culture-positive, and high mean transport were positively associated with cardiovascular events (*p* < 0.05), while serum calcium and serum albumin were negatively associated with cardiovascular events (*p* < 0.05).

Multivariate stepwise COX regression analysis revealed that age (≥65year) (*p =* 0.034*)*, history of CVEs (*p =* 0.010), alkaline phosphatase (*p* < 0.001), and culture-positive (*p =* 0.047)were positive for cardiovascular events, while serum albumin (*p =* 0.032) was negative for cardiovascular events (Table 2).

**Table 2.** Results of univariate and multivariate Regression Analysis.

Variable	Univariate analysis		Multivariate analysis	
	HR (95% CI)	*P*	HR (95% CI)	*P*
Age	1.071 (1.044, 1.100)	<0.001	——	——
Age (≥65 years)	4.298 (2.330, 7.928)	<0.001	2.453 (1.071, 5.619）	0.034
SBP	1.011 (1.001, 1.022)	0.036	——	——
History of CVEs	2.972 (1.738, 5.082)	<0.001	2.296 (1.220, 4.321)	0.010
Calcium	0.201 (0.068, 0.597)	0.004	——	——
Serum albumin				
<30	ref			
30–34.9	0.762 (0.427, 1.360)	0.357	——	——
≥35	0.239 (0.111, 0.514)	<0.001	0.396 (0.170, 0.924)	0.032
Alkaline phosphatase	1.003 (1.002, 1.005)	<0.001	1.004 (1.002, 1.005)	＜0.001
Culture-positive	2.770 (1.308, 5.866)	0.008	2.173 (1.009, 4.682)	0.047
High mean transport	2.396 (1.021, 5.621)	0.045	——	——

SBP: systolic blood pressure; CVEs: cardiovascular events; HR: hazard ratio; CI: credible interval.

### 3.3. Prediction model of CVE

**Figure 2.**
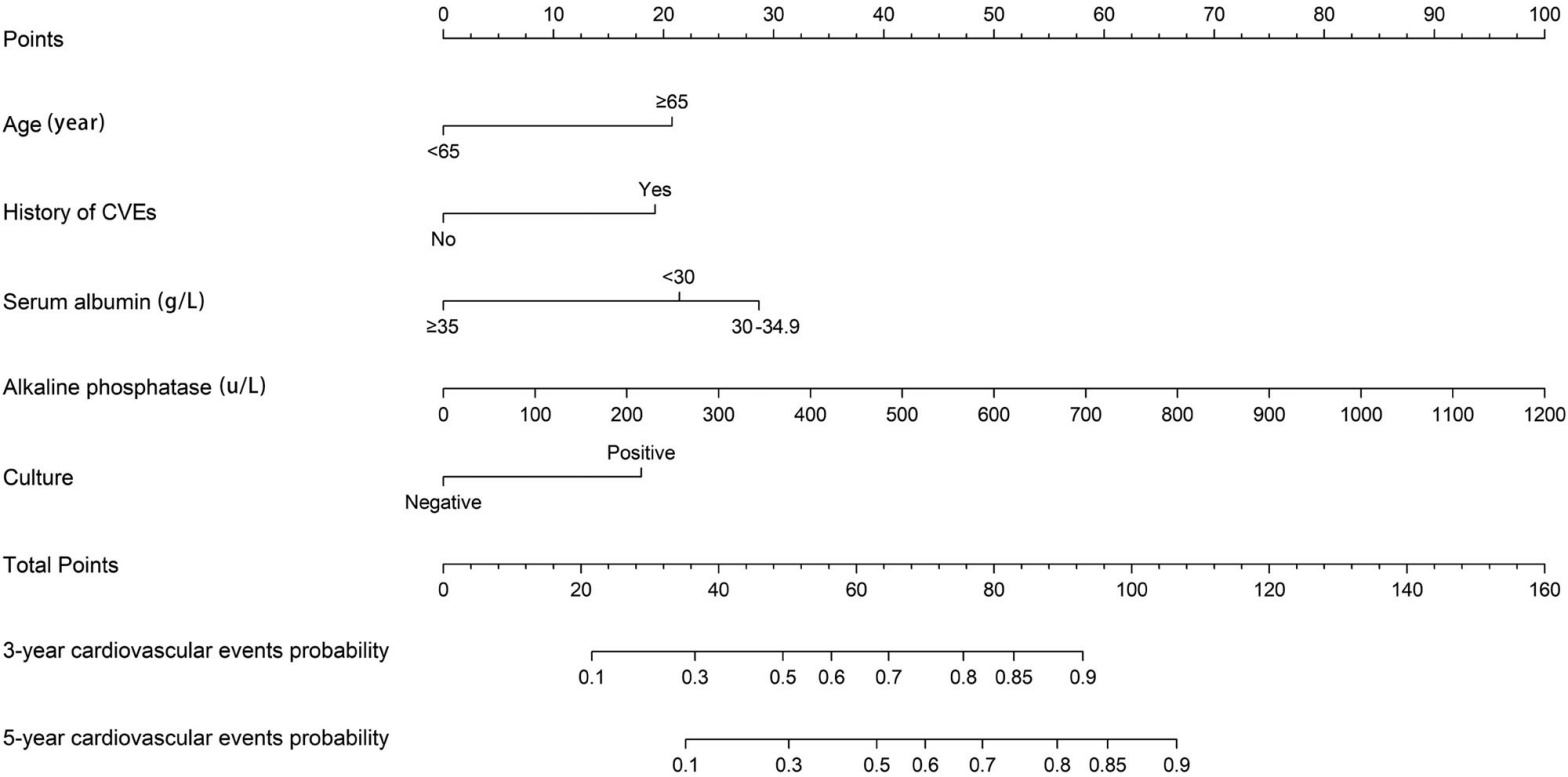
A nomogram for predicting the risk of cardiovascular events in PD patients with peritonitis.

In a multivariate COX regression analysis, we establish a series of consecutive models (Table 3). Along with clinical data, we constructed a nomogram based on the minimum value of the Akaike Information Criterion and Bayesian Information Criterion (Figure 2). The C-index of the final model is 0.732. The variance inflation coefficient was used to evaluate the variables, and the results showed that there was no collinearity in the final model (Table 4). The Somers’ D correlation was −0.463 (*p* < 0.05), demonstrating a well-calibrated model between the observed and predicted probability of cardiovascular events in patients with PD-related peritonitis.

**Table 3.** Multivariate COX regression model for predicting cardiovascular events in patients with peritoneal dialysis-related peritonitis.

Variable HR (95% CI)	Final model	Model 1	Model 2	Model 3	Model 4
Age (≥65 years)	2.453 (1.071, 5.619)	4.442 (2.368, 8.332)	4.526 (2.321, 8.824)	4.103 (1.840, 9.151)	4.835 (2.561, 9.128)
Gender		0.876 (0.497, 1.544)	1.102 (0.586, 2.070)	0.915 (0.494, 1.695)	0.844 (0.477, 1.495)
History of CVEs	2.296 (1.220, 4.321)				
Diabetes			1.687 (0.865, 3.291)	1.836 (0.877, 3.844)	
Smoking			2.165 (0.828, 5.663)		
SBP			1.009 (0.998, 1.021)		
BMI (≥28)				1.500 (0.606, 3.711)	
CRP				1.000 (0.994, 1.006)	
Alkaline phosphatase	1.004 (1.002, 1.005)			1.003 (1.002, 1.005)	1.004 (1.002, 1.005)
Serum albumin	0.396 (0.170, 0.924)			0.461 (0.185, 1.151)	
HDL-C			0.489 (0.198, 1.209)		
Culture-positive	2.173 (1.009, 4.682)				
C-index	0.732	0.605	0.668	0.723	0.639
C-index (Se)	0.033	0.040	0.045	0.036	0.046
AIC value	441.209	463.719	450.284	450.314	456.792
BIC value	453.361	467.770	462.328	472.395	462.868

HR: hazard ratio; CI: credible interval; CVEs: cardiovascular events; SBP: systolic blood pressure; CRP: C-reactive protein; HDL-C, high density lipoprotein cholesterol; AIC: Akaike Information Criterion; BIC: Bayesian information criterion; Se: standard error.

**Table 4.** The collinearity of the final multivariable model.

	GVIF	Df	GVIF (1/(2 × Df))
Age (≥65 years)	1.818	1	1.348
History of CVEs	1.373	1	1.172
Serum albumin (g/L)	1.452	2	1.098
Alkaline phosphatase(u/L)	1.123	1	1.060
Culture-positive	1.029	1	1.014

CVEs: cardiovascular events; GVIF: generalized variance inflation factor; Df: degree of freedom.

### 3.4. Performance of the prediction nomogram in the dataset

**Figure 3.**
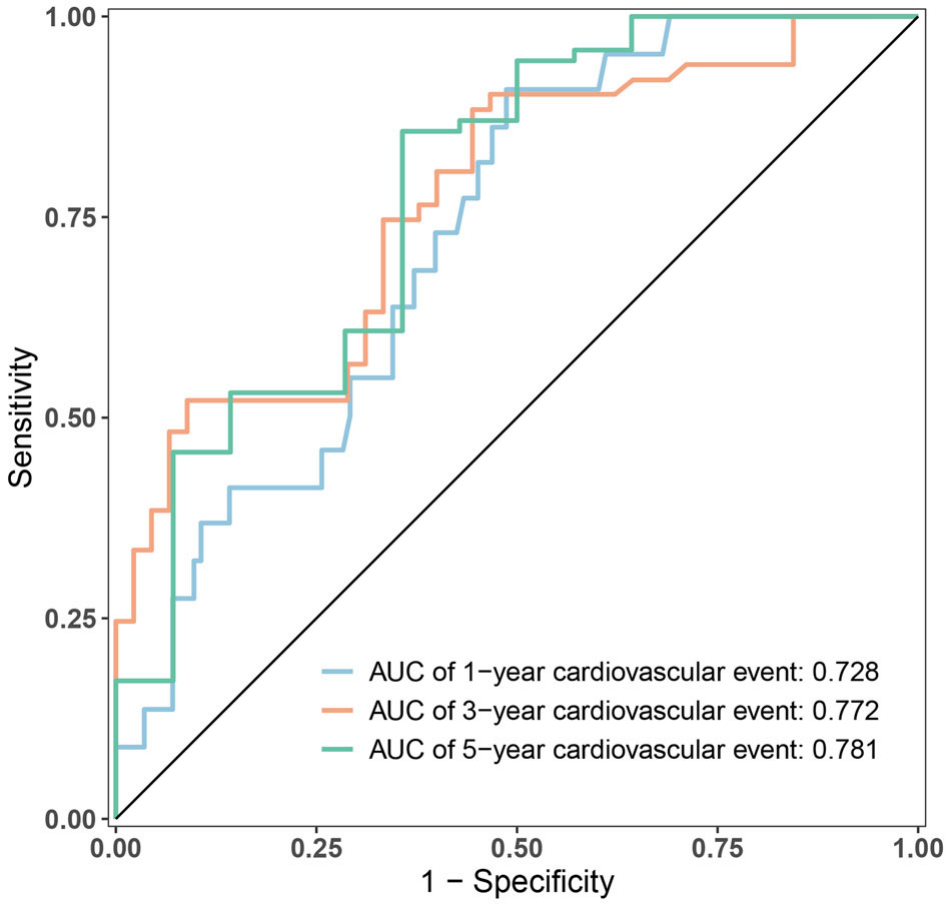
ROC curve for time-dependent cardiovascular events in PD patients with peritonitis.

**Figure 4.**
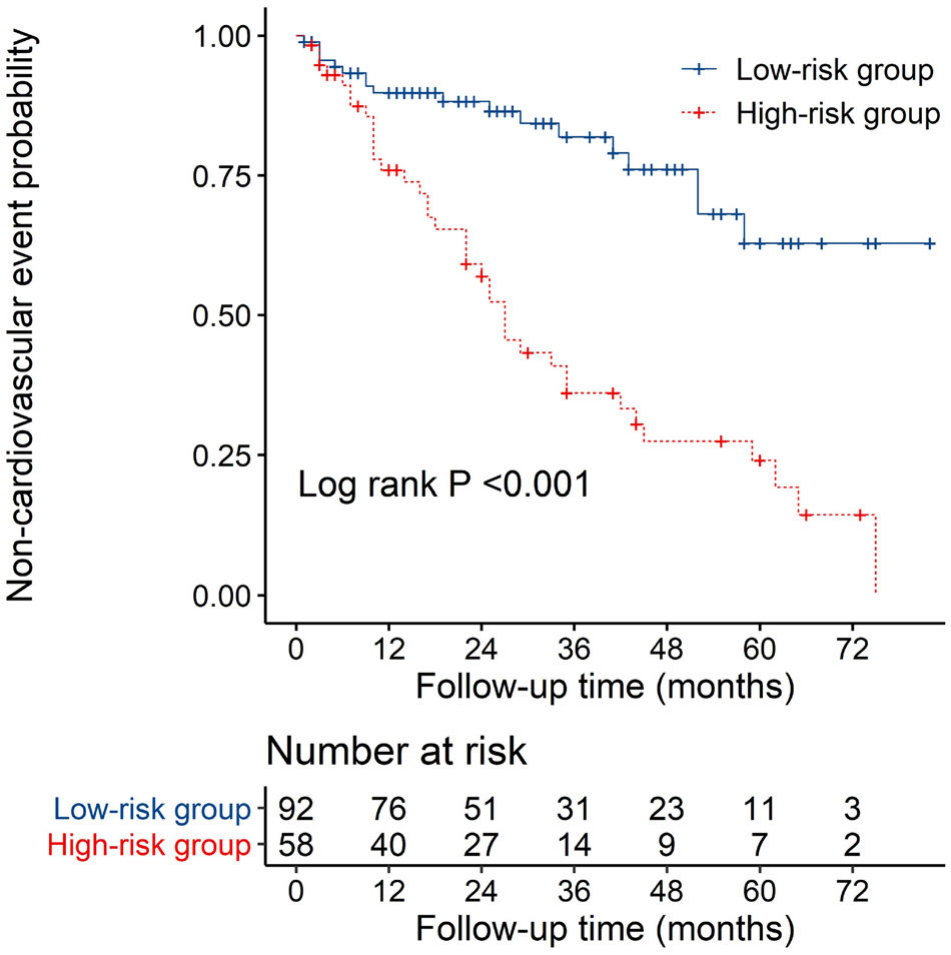
Kaplan-Meier survival curves on the basis of the nomogram.

**Figure 5.**
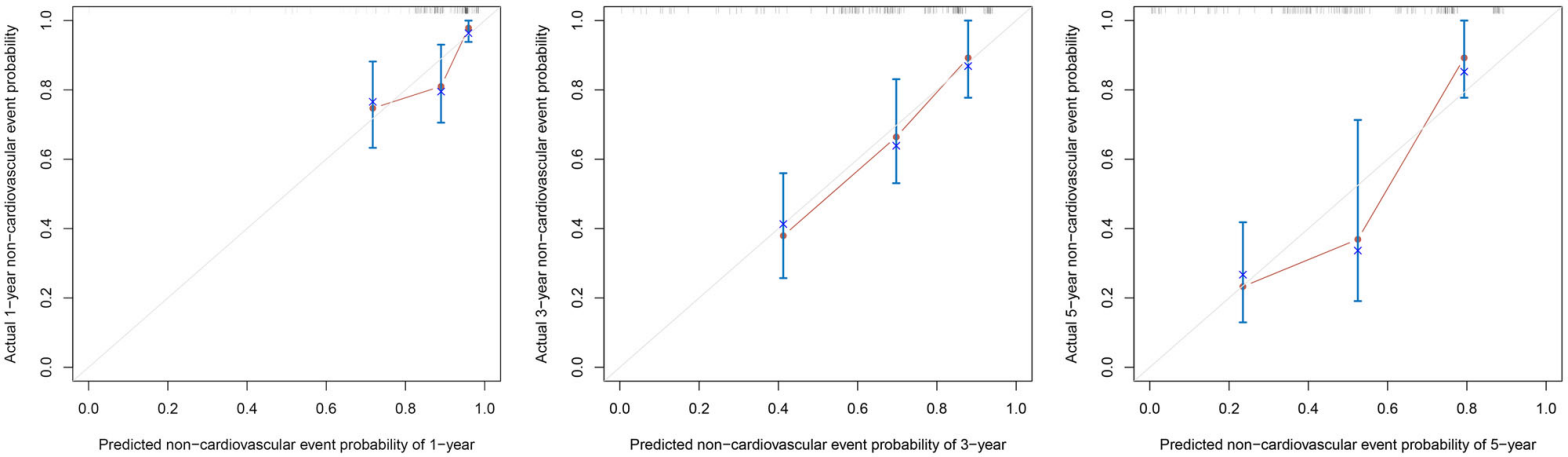
Time-dependent non-cardiovascular events for plots depict the calibration of the nomogram in terms of the agreement between predicted and observed.

The nomogram exhibited reliable performance in the prediction of cardiovascular events, with a time-dependent AUC of 0.728(95% CI, 0.607–0.848), 0.772 (95% CI, 0.651–0.893), and 0.781 (95% CI, 0.603–0.959) at 1, 3, and 5 years, respectively (Figure 3). The score for each predictor was shown in Table 5. The cutoff value is 46.71. If the number of points was greater than 46.71, it was grouped into a high-risk group. The Kaplan-Meier curves revealed that patients in the high-risk group had a significantly higher cumulative rate of cardiovascular events (HR 3.862; 95% CI 2.202–6.772; *p* < 0.001; Figure 4). The calibration plot illustrated that the predicted probability of no cardiovascular events within five years showed good discrimination (Figure 5)

**Table 5.** Nomogram score for each indicator.

Variable	Levels	Point
Age (year)	<65	0.00
	≥65	20.75
History of CVEs	No	0.00
	Yes	19.23
Serum_albumin (g/L)	<30	21.42
	30–34.9	28.67
	≥35	0.00
Alkaline_phosphatase (u/L)	100	8.33
	200	16.67
	300	25
	400	33.33
	500	41.67
	600	50
Culture	Negative	0.00
	Positive	17.96

### 3.5. Sensitivity analysis and internal bootstrap validation to test the robustness of the prediction model

To further verify the robustness of the prediction model, we added sensitivity analysis and internal Bootstrap validation. Due to the small sample size of early-onset peritonitis, the results were inaccurate and deleted. The results showed that the subgroups of late-onset peritonitis and patients with complete data were similar to the prediction model and showed good robustness. From the results of the bootstrap resampling, the model also has good robustness and sensitivity (Table 6).

**Table 6.** Sensitivity analysis and Bootstrap internal validation to test the robustness of the prediction model.

Variable HR (95% CI)	Nomogram model (*n* = 150)	Late-onset peritonitis (*n* = 135)	Complete case (*n* = 145)	Bootstrap
Age ≥ 65 years	2.453 (1.071, 5.619)	2.662 (1.193, 5.942)	2.398 (0.995, 5.775)	2.479 (1.007, 6.172)
History of CVEs	2.296 (1.220, 4.321)	2.282 (1.209, 4.310)	2.146 (1.096, 4.201)	2.377 (0.989, 4.563)
Serum albumin (g/L)				
<30		ref		
30–34.9	1.368 (0.683, 2.740)	1.378 (0.680, 2.795)	1.285 (0.636, 2.596)	1.370 (0.677, 2.962)
≥35	0.396 (0.170, 0.924)	0.381 (0.165, 0.882)	0.376 (0.161, 0.875)	0.363 (0.173, 1.078)
Alkaline phosphatase (u/L)	1.004 (1.002, 1.005)	1.003 (1.002, 1.005)	1.003 (1.002, 1.005)	1.004 (0.999, 1.007)
Culture-positive	2.173 (1.009, 4.682)	2.081 (0.959, 4.515)	2.209 (1.022, 4.775)	2.286 (0.892, 5.349)
C-index	0.732	0.736	0.726	0.747 (0.659, 0.778)
C-index (Se)	0.033	0.035	0.034	0.033 (0.028, 0.040)

HR: hazard ratio; CI: credible interval; CVEs: cardiovascular events; Se: standard error.

## 4. Discussion

The current study developed a novel prediction instrument for cardiovascular events risk in PD-associated peritonitis patients using five readily available baseline variables, including traditional cardiovascular risk factors and dialysis-specific factors. The nomogram demonstrated sufficient accuracy and discrimination.

The current study has the following characteristics. First, patients with PD-associated peritonitis that was cured after their first infection was studied. Peritonitis is a common and serious complication in patients with PD, increasing the patients’ mortality and technical failure rates, and affecting patients’ peritoneal function even when cured [16]. Antibiotic use, especially vancomycin, aggravates the loss of residual renal function in patients. All of these leaded to the poor volume control in PD patients and increased the incidence of cardiovascular events. Second, five risk factors were considered, including older than 65 years, a history of cardiovascular events, serum albumin, alkaline phosphatase, and culture-positive. So far, the elderly has been recognized as a risk factor for cardiovascular events [7]. A recent study found that patients with a history of cardiovascular events had elevated markers of cardiovascular events and were more likely to have cardiovascular events than patients with cardiovascular disease [11]. Several studies have shown that hypoalbuminemia is a risk factor for cardiovascular events in peritoneal dialysis patients [8,17]. Hypoalbuminemia was shown to be related to malnutrition, protein loss, and inflammation that promotes atherosclerosis of blood vessels, leading to cardiovascular events. Second, serum albumin can effectively inhibit human endothelial cell apoptosis, and the integrity of the endothelial structure and function of arterial cells is an important condition for preventing atherosclerosis and preventing thrombosis. Alkaline phosphatase (ALP) is a bone turnover marker; high ALP suggests more prominent renal hyperparathyroidism, which may be associated with cardiovascular diseases. There are documents showing that higher total serum alkaline phosphatase levels cause cardiovascular mortality in patients with PD [18]. ALP can also promote vascular calcification by hydrolyzing pyrophosphate in the arterial wall. Furthermore, studies suggested that inflammation indicated by a higher level of CRP or white blood cell counts might be associated with elevated ALP, responsible for higher mortality [19,20]. A multicenter study found that patients with culture-negative peritonitis had fewer complications and a better prognosis than patients with culture-positive peritonitis [21]. This study also found that culture-positive patients were more prone to cardiovascular events, but the mechanism is still unclear, and more research is needed. Finally, our nomogram performed good discrimination.

Interestingly, in univariate analysis, systolic blood pressure, hypocalcemia, and high transition peritoneal function were positively associated with cardiovascular events. First, In a blood pressure intervention trial, control of systolic blood pressure helped reduce cardiovascular events and revealed that patients with a systolic blood pressure ranging from 130 to 139 mmHg had significantly fewer cardiovascular events than those with a systolic blood pressure >140 mmHg (*p* < 0.001) [22]. However, since systolic blood pressure was collected after half an hour of rest after admission to the hospital during the first onset of peritonitis, confounding factors such as inflammation cannot be ruled out. Second, lower corrected calcium levels are associated with a better prognosis among incident dialysis patients. Yamaguchi *et al.* found that undiagnosed hypocalcemia was a risk factor for cardiovascular events on hemodialysis [23]. In this study, serum calcium was included, and the confounding factors including albumin, alkaline phosphatase, PTH, and other factors were not excluded, so it was excluded in multivariate regression. Third, the high transport function of the peritoneum has a fast transport rate for small molecular substances, and the osmotic pressure gradient is maintained for a short period of time; consequently, the removal effect of water is poor, resulting in patients with excessive volume compliance, which were susceptible to complications such as hypertension and heart failure. However, peritoneal transport function is regulated by peritonitis, therefore it is not an independent predictor of cardiovascular events.

Recent studies have found that early-onset peritonitis has a worse prognosis than late-onset peritonitis [24,25]. In this study, due to the small sample size of early-onset peritonitis, no differences were observed in the model to predict cardiovascular events between early-onset peritonitis and late-onset peritonitis. The internal bootstrap validation shows that the model has good sensitivity and robustness.

### 4.1. Clinical application of the prediction model

The prediction model was clinically instructive. How to use the nomogram: each variable in the figure was marked with a scale on the line segment, representing the value range, and the length of the line segment reflects the contribution to the resulting event. The points in the nomogram represent single scores corresponding to different values. The total points can be obtained by summing up the individual points [26]. Patients were divided into high-risk groups and low-risk groups according to the cutoff value. The care strategy for high-risk group patients includes increasing nutrition, improving hypoalbuminemia and anemia, correcting calcium and phosphorus metabolism disorders, reducing serum alkaline phosphatase, paying attention to the aseptic operation of peritoneal dialysis, avoiding the occurrence of peritonitis, which to decrease the score and turn the high-risk group into a low-risk group, then prevent cardiovascular events in peritoneal dialysis patients.

This study has some advantages. First, this study establishes a model for predicting cardiovascular events in patients with peritonitis for the first time. Second, the outcome events of the model in this study are relatively single, and there are no competing risk events.

This model also has some limitations. First, the sample size of this study was small and no external verification was performed, so the significance of clinical guidance was reduced. Second, it is a retrospective study, there was inevitable recall bias and selection bias. Third, the observed indicators were limited, as brain natriuretic peptide, left ventricular function, coronary CT, and other cardiac function assessment indicators were excluded from the study. In addition, multiple comorbid conditions (i.e., frailty and cognitive impairment), nutritional status, malnutrition–inflammation complex syndrome (MICS), and some medication use are likely to enrich the estimate of the effects of cardiovascular events risk. It seems to be the potential area of bias and uncertainty in this study. Additionally, it may limit the generalizability of the findings. Fourth, age classification may reduce the usage of this model, especially in younger patients.

In conclusion, this study developed a novel nomogram accurately to estimate the risk of cardiovascular events in PD patients with peritonitis within five years. However, the current nomogram model requires external data for validation prior to clinical application.

## Supplementary Material

Supplemental Material

## Data Availability

The data presented in this study are available on request from the corresponding author.
