# Supplementary material for: A nomogram predicts cardiovascular events in patients with peritoneal dialysis-associated peritonitis
Source: Ren Fail. 2022 Sep 26;44(1):1558–67. doi: 10.1080/0886022X.2022.2126785 (PMC9518274; doi:10.1080/0886022X.2022.2126785)
Supplement: Supplemental Material [file IRNF_A_2126785_SM9415.pdf]

| Variable | Original | BootBias | BootSE | BootMed | 95%CI |
|---|---|---|---|---|---|
| age | | | | | |
| <65(year) | ref | | | | |
| ≥65(year) | 0.897 | 0.011 | 0.473 | 0.908 | 0.908 (0.007, 1.820) |
| History of CVE | | | | | |
| 0 | ref | | | | |
| 1 | 0.831 | 0.035 | 0.381 | 0.866 | 0.866 (-0.011, 1.518) |
| Serum albumin | | | | | |
| <30g/L | ref | | | | |
| 30-34.9g/L | 0.313 | 0.002 | 0.384 | 0.315 | 0.315 (-0.390, 1.086) |
| ⩾35g/L | -0.926 | -0.086 | 0.469 | -1.012 | -1.012 (-1.753, 0.075) |
| Alkaline phosphatase | 0.004 | 0.000 | 0.002 | 0.004 | 0.004 (-0.001, 0.007) |
| culture | | | | | |
| 0 | ref | | | | |
| 1 | 0.776 | 0.051 | 0.462 | 0.827 | 0.827 (-0.114, 1.677) |
| C-index | 0.732 | 0.015 | 0.032 | 0.747 | 0.747 (0.659, 0.778) |
| C-index (Se) | 0.033 | 0.000 | 0.003 | 0.033 | 0.033 (0.028, 0.040) |

Supple table 1 Bootstrap resampling 1000 times for internal validation of the model of the C-index
